# Current State of Topical Antimicrobial Therapy in Management of Early Childhood Caries

**DOI:** 10.1155/2014/762458

**Published:** 2014-02-19

**Authors:** JayaBaarathi Jayabal, Ramakrishnan Mahesh

**Affiliations:** Department of Pedodontics and Preventive Dentistry, Saveetha Dental College, Saveetha University, Chennai, Tamil Nadu 600077, India

## Abstract

The treatment of early childhood caries can have a significant economical burden and treatment relapses are frequent. Effective intervention by means of topical antimicrobial agents can reduce the burden of early childhood caries. The main aim in prevention and treatment should focus on inhibition of the growth of oral bacteria. This is a comprehensive review of the literature on the various antimicrobial agents which are proven to be effective in management of this carious progression. The review identified that there is a significant data to suggest use of antimicrobial agents in management of early childhood caries. Antimicrobial agents aid in better management of patients with early carious lesion. The relapse rates are less, when the treatment is combined with the use of antimicrobial agent.

## 1. Introduction 

Early childhood caries (ECC) also known as baby bottle caries, baby bottle tooth decay, and bottle rot is a disease characterized by severe decay in the teeth of infants or young children. This begins as soon as the teeth erupts and can rapidly progress to extensive decay of all primary teeth [1].

Frequent consumption of liquids containing fermentable carbohydrates (e.g., milk, formula, juice, soda, etc.) increases the risk of dental caries which results from prolonged contact between sugars in the liquid and cariogenic bacteria in the teeth. Improper feeding practices without appropriate preventive measures can also lead to this distinctive pattern of caries in susceptible infants and toddlers. The microbial flora mainly involved in ECC is *S. mutans* (MS) [1, 2]. Caries is mainly formed by the accumulation of salivary proteins and adhesive glucans on the enamel surface. Which allow the attachment of *S. mutans* onto the teeth surfaces. These bacteria produce lactic acid during the carbohydrate metabolism which can cause demineralization of enamel surface and cavitation of tooth surface. With sufficient time, the cariogenic microorganisms in the presence of fermentable carbohydrates can alter the critical pH which can induce demineralization of tooth substance, and progress to loss of tooth structure or cavitations [1–3].

The major reservoir from which infants acquire *S. mutans* is their mothers commonly termed as Window of Infectivity and/or also from other primary caregivers [2, 3]. This period is believed to be during the time that teeth are erupting, from seven or eight months until 36 months. However, a single treatment of mothers with an oral antibacterial may not be sufficient to prevent mutans streptococci transmission and subsequent caries in children [3]. Likewise, antibacterial treatment in children may not be sufficient to reduce infection if maternal *S. mutans* levels remain high [1].

There are many risk factors associated with early childhood caries [4]. In addition to the establishment of oral flora, infants and younger children have other unique risk factors for carries, including development of poor dietary habits and food preferences. High-risk infants include those with early signs of ECC, poor oral hygiene (of both infant and mother), limited exposure to fluoride, and frequent exposure to sweet beverages [5]. However, by the time the mandibular and maxillary primary incisors erupt, bottle or breast feeding is supplemented by a variety of solid foods. These foods include starches and other sucrose-containing foods with cariogenic potential, which provide an improved substrate to promote the proliferation of cariogenic bacteria. In addition, adding a sweetener in bottles fed to infants is a major risk factor for early caries [6, 7].

The effects of early childhood caries may in many children extend beyond pain and infection. It also affects the child's ability to eat, communicate, and learn. Pulpal pain associated with deep caries lesions can lead to reduced quality of life in children. Any alternation or interruption in play and school work from pain due to caries can induce emotional stress, including anger and instability. In a prescholar as a result of aesthetic and/or phonetics problems, children may be teased by other children, which could negatively affect their self-esteem [1, 8].

## 2. The Need for Prevention of Early Childhood Caries

Primary prevention of dental disease not only preserves healthy teeth but also decreases the current tremendous demand for restorative and surgical care. There are several antimicrobial agents available for the management for early childhood caries. As the most commonly used preventive agents in management of ECC include application of fluoride gels, fluoride varnish, sealants, chlorhexidine varnish, 10% povidone iodine, and xylitol oral syrup. ECC prevention should start during the prenatal period, progress through the perinatal period, and continue with the mother and infant within the context of the family and during preschool programs [9, 10]. Given the evidence for vertical transmission of cariogenic bacteria, primarily from mother to child, involving pregnant women in oral health screening, dental treatment, and education on oral health hygiene and bolstering their nutrition along with the use of fluoride toothpaste are strategies that can assist in the prevention or delay of ECC [9]. Prenatal visits may also provide an opportunity to build awareness of the importance of oral health for mothers and their infants [11].

With ECC being a result of the interplay of oral bacteria, substrate, and host as well as family, economic, and social conditions, health-promotion strategies that emphasize community development and address the determinants of health are required along with strategies that focus on disease prevention [12, 13]. There are various commercially available agents which are proven to have antibacterial properties against *S. mutans*. The current review highlights the various agents available with their mechanism of action and their advantages.

## 3. Fluoride Varnish and Gels

Fluorides are the most commonly applied topical agents in management of early carious lesions. Topical fluorides have been found to be effective in preventing caries. A Cochrane systematic review has concluded that, on average fluoride varnish reduced caries in the primary dentition by 33% [14]. They are traditionally applied as fluoride mouth rinses, concentrated fluorides on trays, fluoride varnishes and fluoride tooth pastes. Fluoride varnish plays an important role in prevention of caries because of its ease of application. The commercially available fluoride varnish includes Duraphat, Duraflor, and Flor protector. There are various studies proposed on mechanism of action of fluoride on oral bacteria. Fluoride inhibits the glycolytic enzyme enolase, in a quasi-irreversible manner [15]. Direct action seems also to occur in inhibition of heme-based peroxides with binding of fluoride to heme. The flavin-based peroxides of many oral bacteria are insensitive to fluoride [15]. Another mode of action involves formation of metal-fluoride complexes, most commonly AlF4-. These complexes are responsible for fluoride inhibition of proton-translocating F-ATPases and are thought to act by mimicking phosphate to form complexes with ADP at reaction centers of the enzymes [15]. Zickert and Emilson proposed that fluorides do not reduce the level of *S. mutans* in saliva but only cause remineralisation of early carious lesion [16].

The risk of acute toxic reactions with the varnishes is minimal compared to APF gels and fluoride tooth pastes. In addition, the risk of dental fluorosis is minimal because children are not frequently exposed to fluoride varnishes, as they are to fluoride supplements. Duraphat is contraindicated in patients with ulcerative gingivitis and stomatitis. Also based on the caries risk of the child the professionals can also recommend use of fluoride dentifrices [17]. Professional applications of fluoride varnish twice/year are effective in reducing caries incidence [18].

Varnishes deliver fluoride to the surface of enamel and to subsurface carious lesions, where it forms deposits of calcium fluoride and provides a reservoir of fluoride ions [19, 20]. Fluoride varnish efficacy in young age group provides additional rationale for an early dental visit, especially for high-caries-risk children, since the application of fluoride varnish at this first visit will help reduce future disease. Although more frequent varnish applications were more beneficial, one application was preferable to none [21].

The use of silver compounds in surgical disinfection has a long history with good clinical results. The combined use of silver diamine fluoride (SDF) has been in use to arrest dental caries in many countries. A 38% (44,800 ppm fluoride ions) SDF solution is commonly used to arrest caries in primary teeth of children [22]. Topical application of SDF to arrest dental caries is a noninvasive procedure that is quick and simple to use. The possible disadvantage of using SDF is that it stains the carious teeth and turns the arrested caries black. It also has an unpleasant metallic taste that is not liked by patients, especially children [22, 23].

Rosenblatt et al. 2009 conducted a clinical trial to analyze the efficacy of SDF when compared to fluoride varnish [23]. The results suggest that SDF is more effective than fluoride varnish and may be a valuable caries-preventive intervention. The current literature suggests that SDF can be an effective agent in preventing new caries and in arresting dental caries in the primary teeth of the children. It can be used to arrest caries progression in very young children who are less cooperative, and it allows definitive restoration to be performed when they grow older and become more receptive to dental procedures [22, 23]. The mechanism of action of SDF is unique because it is able to act in two ways. The silver component acts as an antibacterial and prevents the formation of new biofilm at the application site. The silver and the fluoride then work together to form fluorapatite, strengthening the tooth's outer surface.

## 4. Chlorhexidine Varnish

For appropriate caries prevention an antibacterial that also attacks effectively the lactobacilli species that are strongly related to caries progression [24]. Chlorhexidine gel (CHX) is a promising new treatment for the prevention of dental caries. Chlorhexidine has a long history of use in caries prevention trials. Some studies have found it efficacious, while others have not [25].

Chlorhexidine is the component of bisbiguanides. Chlorhexidine reduces MS but does not usually eliminate it except with intensive, high-concentration, and repeated applications [26]. Intervention with multiple applications over periods of months of 0.12% chlorhexidine gluconate can significantly reduce MS levels and in combination with fluoride therapy can markedly lower the risk for future caries. However, the chlorhexidine is much less effective against LB in the mouth.

As an anticaries agent, CHX is used at concentrations ranging from 0.1 to 40 percent in solutions, gels, chewing tablets, and varnishes [27]. A previous meta-analysis of selected studies gave an overall caries inhibiting effect of CHX of around 46 percent [28]. The cation chemical binds well to negatively-charged surfaces including bacterial cell walls, acquired pellicle, plaque layers, and buccal mucosa [29]. As such, it has very good substantivity and hydrophilicity which maximizes its antibacterial effects over an extended period of time [30].

Petti and Hausen showed that CHX gel applications showed moderate anti-MS effect but negligible caries-preventive effect [31]. A 1.0% flavored chlorhexidine gel has been developed by the University Of Iowa College Of Dentistry and has been used directly with children in Phase I FDA-sanctioned trials. This product has the great advantage of being in a toothpaste-like formulation and having a pleasant raspberry flavor that children like. Considering this limitation, it is tentatively concluded that CHX varnish has a moderate caries-inhibiting effect when applied every 3-4 months, but its caries-inhibiting effect seems to have diminished around 2 yr after the last application [25].

## 5. Povidone Iodine

Povidone iodine is used as a topical antimicrobial agent in prevention of dental caries. Based on the study conducted by Simratvir et al. regular applications of 10% povidone iodine application can be a good alternative to control dental caries in children affected with ECC along with oral rehabilitation [32]. Lopez et al. concluded that periodic topical application of an iodine solution to the dentition of children at high risk for ECC should suppress dental *S. mutans* levels and in turn reduce risk for the development of ECC [33]. Bimonthly topical application of a 10% povidone iodine solution to the dentition of babies at high risk for ECC increased disease-free survival [34].

Topical application of a 10% povidone iodine solution to the dentition of infants every 2 months in a double-blind, placebo-controlled clinical trial for 1 year increased the number of caries-free infants [35]. Povidone iodine solution being a water soluble compound liberates iodine which has antimicrobial action. The slow release of iodine from povidone iodine allows for long term antibacterial effect [36].

## 6. Xylitol 

Xylitol is a five carbon sugar alcohol that looks and tastes like sucrose and is therefore a good sugar substitute and its beneficial effect is that it is not metabolized by the cariogenic bacteria and that it has antibacterial properties [37]. Unlike the taste of chlorhexidine, xylitol has sweet taste and also has an antimicrobial activity. Daily xylitol-wipe application significantly reduced the caries incidence in young children as compared with wipes without xylitol, suggesting that the use of xylitol wipes may be a useful adjunct for caries control in infants [38]. According to the clinical trial conducted by Solderling and coworkers for six years mothers who chewed xylitol gum during the first two years of the children's lives had highly significant reductions in colonization of MS, and, very importantly, had dramatically less dental decay five years later [37–39]. The transmission of *S. mutans* was hindered by the xylitol-induced shift from xylitol-sensitive to less adherent xylitol-resistant strains of the caries-associated bacterium [18]. Thus, xylitol has better antimicrobial activity and showed better reduction in *S. mutans* count and maternal transfer from the mother to their children.

Parents of infants and toddlers should be encouraged to reduce behaviors that promote the early transmission of MS. Chewing xylitol-containing gums during the period of primary teeth eruption (6–20 moths) may reduce caries in the primary dentition [18].

## 7. Liquorice 

Another intervention aimed at specifically targeting *S. mutans* is an innovative lollipop (Dr. John's Candies, Grand Rapids, MI, USA) made from the Chinese herb, glycyrrhiza uralensis (liquorice root). Its active ingredients, glycyrrhizol A and B, have demonstrated substantial antimicrobial activity against *S. mutans* [40]. It was postulated that the use of a lollipop as the mode of delivery of the active ingredient (glycyrrhizol A and B) would have a distinct appeal to the children. Administering the lollipop to children required no monitoring; however, observation by parents or caregiver was recommended, to avoid any possible choking or minimizing the wastage of the lollipop due to incomplete consumption by the children.

## 8. Conclusion

ECC is mostly an infectious disease, but its effects can be prevented by early effective interventions. There is sufficient evidence to support and recommend topical applications of antibacterial agents, such as chlorhexidine and povidone iodine, in order to arrest early childhood caries (Table 1). The management often requires education of both the parents and the child to improve their dental awareness and attitude toward dental health.

## Figures and Tables

**Table 1:** Brief overview of various topical antimicrobial agents.

Author Name	Agent used	Comments
Marinho et al. 2013 [14]	Fluoride varnish	Substantial caries inhibiting effect of fluoride varnish in both permanent and primary dentition

Rosenblatt et al. 2009 [23]	Silver diamine fluoride	SDF can be an effective agent in preventing new caries lesion and in arresting dental caries in primary teeth.

Petti and Hausen 2006 [31]	Chlorhexidine varnish	CHX gel showed moderate anti-MS effect but negligible caries preventive effect.

Simratvir et al. 2010 [32]	Povidone iodine	Povidone iodine application can be a good alternative to control caries in children affected with ECC.

Zhan et al. 2012 [38]	Xylitol wipes	7 times fewer children from the xylitol-wipe group developing new caries compared with the placebo group

He et al. 2006 [40]	Liquorice	Glycyrrhizol A and B in liquorice have demonstrated substantial antimicrobial activity against *S. mutans *
